# Can Paracorporeal Bi-VAD Improve the Outcome of a Patient Candidate for Heart Transplant?

**DOI:** 10.1155/crit/5080204

**Published:** 2025-12-04

**Authors:** Giuseppe Fischetti, Lorenzo Giovannico, Federica Mazzone, Nicola Di Bari, Domenico Parigino, Massimo Padalino, Tomaso Bottio

**Affiliations:** Cardiac Surgery Unit, Department of Precision and Regenerative Medicine and Ionian Area (DiMePRe-J), University of Bari, Bari, Italy

## Abstract

**Background:**

Postcardiotomy cardiogenic shock (PCCS) is a critical condition with high mortality. When biventricular dysfunction occurs, veno-arterial ECMO alone may not provide adequate unloading. Paracorporeal BiVAD can offer balanced support as a bridge to heart transplantation.

**Case:**

A 61-year-old woman developed PCCS following bioprosthetic aortic valve replacement. Despite VA-ECMO and intra-aortic balloon pump support, biventricular failure persisted. A paracorporeal BiVAD was implanted, achieving rapid hemodynamic and pulmonary improvement. After 7 days of BiVAD support, she underwent successful orthotopic heart transplantation. At 1-year follow-up, the patient remains alive without graft rejection or vasculopathy.

**Conclusion:**

Early conversion from VA-ECMO to BiVAD may improve outcomes in refractory PCCS with biventricular failure, serving as an effective bridge to transplantation.

## 1. Introduction

Postcardiotomy cardiogenic shock (PCCS) remains a major clinical challenge with high mortality. Mechanical circulatory support (MCS), including venoarterial extracorporeal membrane oxygenation (VA-ECMO), has been the standard of care in refractory cases. The ease of use and positioning, even at the patient's bed, makes it the gold standard treatment for patients with cardiogenic shock (CS) unresponsive to pharmacological treatment. However, when biventricular dysfunction and pulmonary compromise coexist, VA-ECMO alone may not be sufficient. In this report, we describe a rare case where a paracorporeal biventricular assist device (BiVAD) was employed successfully as a bridge to transplantation. This approach could offer valuable insights into patient selection and individualized MCS strategies. This technique could be useful in patients with the same conditions and demonstrates how custom-made short mechanical support (ST-MCS) can be used to reduce risks and maximize benefits.

## 2. Case Presentation

We report the case of a 61-year-old woman who was followed up for severe aortic insufficiency (EROA: 0.30 cm^2^) with initial signs of left ventricular dilatation (LV diastolic diameter 58 mm). She underwent elective cardiac surgery. A bioprosthetic aortic valve replacement was performed with the aid of a ministernotomy. Cardiac arrest was obtained with an antegrade selective cardioplegia administration. PCCS occurred, requiring VA-ECMO assistance. A mixed central-peripheral ECMO configuration was preferred (arterial cannula in aorta and venous cannula at groin). IABP 1:1 was added for venting. The post-op coronary angiography revealed a right coronary dissection, promptly treated with a double stenting procedure. In the following days, the attempt to wean from mechanical assistance (VA-ECMO and IABP 1:1) was ineffective and treatment with continuous renal replacement therapy (CRRT) for oliguria was initiated.

**Figure 1:**
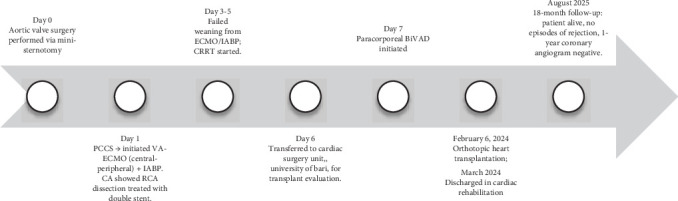
Clinical timeline summarizing the key stages of the patient's course from initial surgery to heart transplantation and follow-up.

The patient was transferred to the Cardiac Surgery Unit of the Bari Policlinic (Italy) for evaluation of possible inclusion in the emergency heart transplant list. Upon arrival the patient was mechanically ventilated (MV) responsive to simple orders, in V-A ECMO at 4.5 L/min and IABP 1:1, with dobutamine infusion 5 *μ*m/kg/min and adrenaline 0.07 *μ*m/kg/min. Chest radiography showed congestion of the lung fields with right pleural effusion (Figure 1). Transthoracic echocardiography (TEE) revealed severe biventricular dysfunction (EF, 15%; TAPSE, 10 mm). Chest X-ray, TEE, laboratory tests, antibodies reaction panel (ARP), and the patient's clinical conditions did not contraindicate inclusion on the national emergency heart transplant list. Due to worsening of the parenchymal condition and pulmonary microcirculation, we decided to upgrade the mechanical assistance with a paracorporeal Bi-VAD. In addition to biventricular support, particular attention was paid to the need for effective left ventricular (LV) venting. Inadequate unloading of the LV during VA-ECMO can lead to pulmonary edema, increased myocardial oxygen demand, and LV thrombus formation.

**Figure 2:**
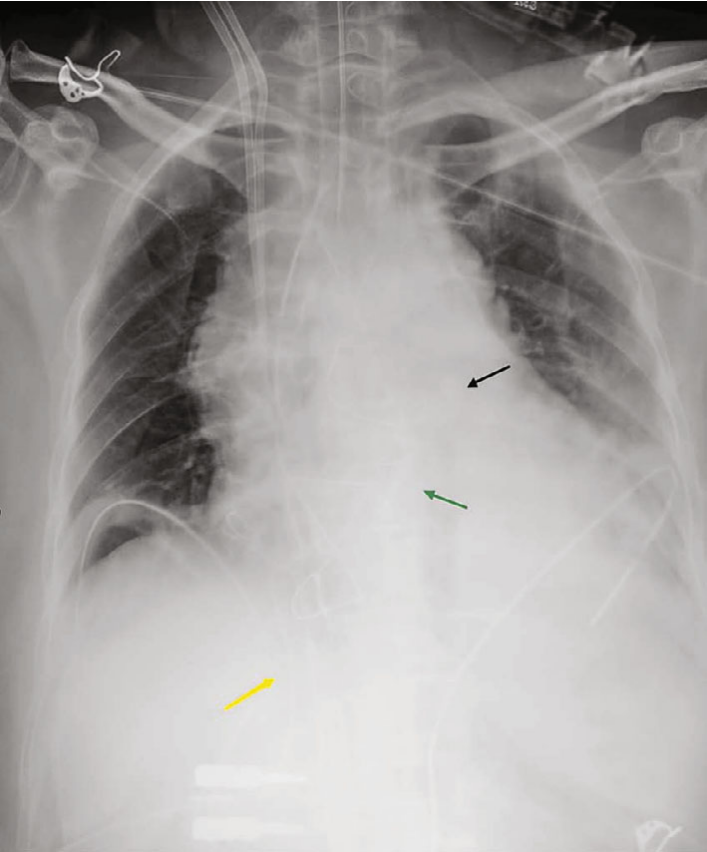
Chest X-ray upon arrival. Black arrow: intra-aortic balloon pump (IABP); yellow arrow: drainage venous cannula; and green arrow: bioprosthetic valve.

**Figure 3:**
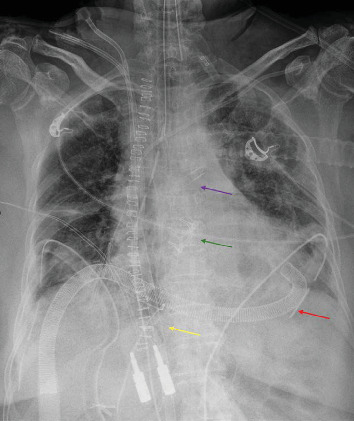
Chest X-ray 2 days after BiVAD placement showing partial improvement of pulmonary edema. Red arrow: inflow cannula of the LVAD (from the apex to the pump); yellow arrow: inflow cannula of the RVAD (from the inferior vena cava to the pump); purple arrow: outflow cannula of the RVAD (from the pump to the pulmonary artery); green arrow: bioprosthetic valve.

The inflow cannula for the LVAD (from the LV apex to the pump) was placed through the fourth intercostal space of the left chest wall. The outflow cannula (from the pump to the patient) was connected to the right femoral artery. The RVAD outflow cannula (from the pump to the patient) was inserted into the main pulmonary artery through the second intercostal space, with the inflow cannula (from the patient to the pump) connected to the preexisting venous ECMO cannula (Figure 2). The flows of the two systems were uniform and balanced to ensure hemodynamic stability. The system was based on extracorporeal centrifugal pumps equipped with an integrated heat exchanger and oxygenator, ensuring both circulatory and respiratory support during BiVAD assistance. Chest X-ray showed partial resolution of pulmonary edema after 2 days of biventricular mechanical assistance (Figure 3). In our approach, the placement of the apical LV cannula ensured direct and continuous venting. We opted for femoral cannulation for the outflow of the paracorporeal pump rather than reusing the ascending aortic cannula previously employed during VA-ECMO. This choice was guided by both technical and clinical considerations. Reopening the central cannulation site posed a risk of bleeding, while femoral access allowed for a more streamlined setup. More importantly, femoral cannulation enabled early sternal closure, a key step in reducing the risk of mediastinitis and other sternal wound infections. This approach facilitated safer BiVAD management and optimized conditions for eventual heart transplantation. The patient underwent successful orthotopic heart transplantation in February 2024 following 7 days of BiVAD support.

A summary of the patient's clinical course is presented in Figure 1.

**Day 0 (January 30, 2024):** The patient developed PCCS following aortic valve replacement and initiation of VA-ECMO and IABP support.

**Day 7 (February 6, 2024):** The patient underwent successful orthotopic heart transplantation after 7 days of paracorporeal BiVAD support.

Since transplantation, the patient has remained alive and in good clinical condition. Serial endomyocardial biopsies have consistently shown no evidence of acute or chronic rejection, and coronary angiography has demonstrated no signs of graft vasculopathy. As of the time of writing (November 2025), she continues to do well, 21 months after heart transplantation.

## 3. Discussion

This case highlights the clinical challenges of managing PCCS complicated by severe biventricular failure. While VA-ECMO remains the first-line option for temporary circulatory support, it may not provide sufficient ventricular unloading, particularly in the presence of compromised pulmonary compliance. Although VA-ECMO is a well-established bridge to decision or recovery, it can induce LV distension, pulmonary congestion, and increased myocardial oxygen demand when used in isolation. These effects are especially concerning in patients with depressed biventricular function and compromised pulmonary compliance. Alternative strategies for LV venting, such as atrial septostomy, transseptal cannulation, or the use of percutaneous devices like the Impella (ecpella configuration), may also be considered based on patient anatomy and device availability. In this case, the surgical BiVAD configuration provided effective unloading and contributed to rapid pulmonary recovery.

Furthermore, the ProtekDuo and IMPELLA system was not immediately available, and the patient's condition was too unstable to delay mechanical support initiation. For these reasons, we proceeded with the implantation of a custom paracorporeal BiVAD, allowing direct and balanced decompression of both ventricles and rapid resolution of pulmonary edema.

As extensively reviewed by Donker et al., failure to actively unload the LV during VA-ECMO is associated with worse outcomes, and a wide array of percutaneous and surgical strategies—including BiVAD—may be considered based on clinical urgency, vascular access, and biventricular involvement [1].

As in the case described, due to poor LV-RV function, ECMO support may cause LV distention leading to myocardial ischemia, LV thrombogenesis, pulmonary edema due to backpressure, and pulmonary thrombosis [2]. Effective unloading of the left ventricle is essential to reduce pulmonary congestion, which Schrage et al. [3] have identified as a key prognostic factor in patients supported with VA-ECMO. Persistent pulmonary overload not only impairs gas exchange but also contributes to right ventricular strain and systemic hypoperfusion. Moreover, Pagani et al. highlighted that severely reduced pulmonary compliance is independently associated with poorer outcomes during mechanical support, reinforcing the importance of early and tailored decompression strategies in biventricular failure [4]. Furthermore, some authors have emphasized the importance of appropriately timing unloading both ventricles for successful bridging to recovery, and an early Bi-VAD application strategy would lead to better outcomes in terms of successful bridging to recovery or transplantation, with lower complication and mortality rates than ECMO-treated patients. [5] Moreover, as described by Kawashima et al. and Lorusso et al, adding right ventricular support may offer several physiological advantages, including antegrade pulmonary blood flow, reduction of LV distension, maintenance of pulmonary circulation, and avoidance of complications related to retrograde arterial cannulation [6, 7]. The present case supports existing evidence that early transition to biventricular mechanical support may enhance survival compared with delayed conversion strategies [8].

Our experience aligns with these findings, demonstrating that prompt BiVAD support could be a lifesaving bridge to transplant in patients with complex biventricular failure, especially when conventional ECMO strategies are not feasible.

## 4. Conclusion

Paracorporeal BiVAD could be an effective support in a patient with severe biventricular failure post-cardiotomy, enabling stabilization and successful heart transplantation. This case underscores the value of individualized MCS strategies and the potential role of BiVAD in bridging to transplant when conventional ECMO options are not feasible.

## Nomenclature

VA-ECMO: venoarterial extracorporeal membrane oxygenation
IABP: intra-aortic balloon pump
LVAD: left ventricular assist device
RVAD: right ventricular assist device
BiVAD: biventricular assist device
CRRT: continuous renal replacement therapy
PCCS: postcardiotomy cardiogenic shock
